# Frailty and polypharmacy among geriatric home care patients: an epidemiological analysis and implications for optimised care

**DOI:** 10.1186/s12912-025-03850-y

**Published:** 2025-09-26

**Authors:** Khalid Abdullah Aljohani

**Affiliations:** Community Health Nursing Department, Nursing College Taibah University, Al Madinah, Saudi Arabia

**Keywords:** Geriatric assessment, Home care, Frail older adult, Polypharmacy

## Abstract

**Background:**

The ageing population is an international phenomenon. This study investigates the epidemiology of geriatric home care patients in Madinah, Saudi Arabia, focusing on frailty and polypharmacy.

**Methods:**

A cross-sectional descriptive design was utilised to assess the profiles of home care geriatric patients based on their comprehensive geriatric assessment (CGA) forms from the medical records of the Home Care Administration. Sampling used a census approach involving all eligible CGA forms during the study period.

**Results:**

The sample included 281 patients, predominantly female, with a mean age of 79 years. The logistic regression models for frailty and polypharmacy demonstrated acceptable predictive accuracy and good model fit. Significant predictors of a higher likelihood of frailty were increased age, depression, and diabetes. In the polypharmacy model, significant predictors are other chronic conditions, medical complications, depression, and hypertension, all of which increase the likelihood of polypharmacy.

**Conclusions:**

The high prevalence of both conditions reflects the substantial clinical burden in this population, emphasising the complexity of their care needs. Moreover, the study recommends that future research explore universal operational definitions of frailty in national practice to better align with global standards.

**Clinical trial number:**

Not applicable.

## Background

According to the World Health Organization (WHO), 1.4 billion people were older than 60 years in 2020. This number is projected to double by 2050, with those aged 80 years and older estimated to reach 426 million [1]. From a healthcare perspective, the ageing population is an international phenomenon. It increases demands on healthcare systems, results in a greater need for healthcare providers, escalates operational costs, and increases the financial strain on healthcare systems [2, 3].

Healthcare systems worldwide have designed both reactive and proactive approaches to mitigate the risks posed by the growing healthcare needs of the ageing population. These include transitional care models that aim to reduce readmissions and provide continuous care in supportive settings, including nursing facilities and patients’ homes [4, 5]. From an epidemiological standpoint, prior studies in Saudi Arabia show that the prevalence of diabetes mellitus (DM) among older adults is 55%, hypertension (HTN) is 49%, and the co-occurrence of DM and HTN is 27%. Additionally, the prevalence of depression and urinary incontinence was 6%, while polypharmacy was reported at 25% [6]. Chronic diseases were evident in another study, which found that they affected 60% of the geriatric population, with HTN at 58% and DM at 49% [7]. Overall, the high prevalence of chronic diseases among the Saudi geriatric population has contributed to challenges related to polypharmacy [8].

Polypharmacy further complicates the health of older people because of alterations in pharmacokinetics and pharmacodynamics [9]. It is a significant challenge, affecting 55% of Saudis older than 60 years, with an average of 6.4 prescribed medications [10, 11]. The global prevalence of polypharmacy ranges from 30% to more than 70%, influenced by age, setting, and comorbidity burden [6]. A systematic review of 54 studies revealed a pooled polypharmacy prevalence of 37% [12]. A detailed pooled prevalence showed that polypharmacy was evident in Europe (46%), Oceania (45%), North America (41%), Asia (29%), and South America (28%) [13].

The physiological changes of frailty contribute to widespread disruptions in bodily functions and increase an individual’s risk of disease, social isolation, and mortality. Using Fried’s frailty phenotype, Alqahtani and colleagues estimated the prevalence of frailty among older Saudi individuals to be 21.4% [14]. Additionally, a systematic review and meta-analysis focusing on Middle Eastern countries reported a wide range of frailty prevalence rates among individuals aged 60 and older, with figures such as 37% in Saudi Arabia, 47% in the United Arab Emirates, 71% in Egypt, 47% in Iran, 28.7% in Turkey, and 36% in Lebanon [15]. Globally, frailty prevalence varies widely depending on the setting and assessment tools [16, 17]. Physical frailty was higher in females (15%) than in males (11%) in a review of 62 countries [18].

In general, polypharmacy may cause negative health consequences, including overprescribing, leading to potential adverse effects, non-adherence, and increased patient safety risks [8, 19]. Similarly, frailty increases older patients’ vulnerability, raising the risk of morbidity and mortality [20]. Therefore, assessing the epidemiological profile of the geriatric population is essential for better healthcare service planning and utilisation. This study aims to conduct an epidemiological analysis of geriatric homecare patients in Madinah, Saudi Arabia, and to identify frailty and polypharmacy risk factors among CGA components. The study focuses on the rapidly growing geriatric population in Saudi Arabia and its specific examination of frailty and polypharmacy risk factors among homecare patients in Madinah. With an ageing population, the demand for effective and targeted healthcare strategies that address the complex health needs of older adults is increasing. This study may offer insights that can help guide healthcare policymakers, providers, and caregivers in optimising care for this vulnerable population.

## Methods

### Study design, participants, and data collection

A retrospective cross-sectional study design was utilised to assess the profiles of home care geriatric patients based on their medical records data at the Home Care Administration in Madinah, Saudi Arabia, from August to November 2022. Census sampling was employed by including all available comprehensive geriatric assessment (CGA) forms from homecare patients enrolled for ≥ 1 year. The rationale for excluding those with ≥ 1 year was to ensure stability of the homecare service delivery where the patient became fully involved in the care provision. Data were collected by homecare nurses who are authorised to access patients’ medical records during their daily activities. Therefore, no bias could be involved. The CGA forms of patients receiving home care for more than a year were included. Since all CGA forms were completed and saved in the patients’ medical records, no patient records were excluded. Data were extracted into a summary form, including all necessary variables. Checking a random sample of summary forms was undertaken by the charge nurse to maximise summary form reliability. This retrospective chart review study was approved by the Institutional Review Board. As such, informed consent was waived due to the use of de-identified secondary data. The study was conducted in accordance with the guidelines and the Declaration of Helsinki.

### Sample size

The sample size was calculated according to the event-per-variable heuristic rules of thumb (*n* = 100 + 50i), where ‘i’ represents the number of independent variables [21]. The formula was used as a conservative guide to ensure that subgroup analyses could be reasonably powered, especially given the lack of prior prevalence data specific to our population. Importantly, the actual sample size of 281 was not the result of sampling but rather a full inclusion (census) of all eligible CGA records during the study period. This approach ensures full coverage of the homecare geriatric population accessible to the researchers at the time. Given the finite size of the eligible population and the study’s descriptive nature, the sample size is considered acceptable for the study objectives [21].

### Measurement

#### The outcome variables

The dependent variables in this study were frailty and polypharmacy, both of which were derived from the CGA forms. Frailty was operationally defined as age ≥ 85 years, consistent with the criteria used in the CGA forms currently implemented by the study location. This approach reflects the practical use of age as a risk stratifier within local service delivery models. However, we acknowledge that this does not align with internationally accepted frailty definitions, such as Fried’s frailty phenotype, which incorporate physical, cognitive, and psychosocial dimensions [22]. Due to constraints of the retrospective study design, potential variability in the completeness of CGA forms, and lack of systematic application of validated scales such as Fried’s phenotype and the Edmonton Frail Scale in the study location CGA forms, the researcher retained the study location’s operational definition of frailty. Furthermore, polypharmacy was operationally defined as the prescription of five or more medications.

### The explanatory variables: demographics and clinical characteristics

Sociodemographic factors include gender, age, and time since admission to homecare services. The clinical characteristics were derived from the CGA. The CGA is a multidimensional assessment of the geriatric population aimed at enhancing their health, quality of life, and independence. It has been implemented in various healthcare settings, including hospitals and primary care practices [22, 23]. It supports decision-making by involving multidisciplinary healthcare teams in assessment [15, 16]. The CGA was utilised at the study location; therefore, data were abstracted using a summary of key findings from the CGA forms. Although the CGA includes several assessments, such as medical, functional, cognitive, social, and nutritional assessments, the current study focuses on the following variables, which were derived from the CGA:

1. Diagnoses of DM, HTN, or both;
2. The presence of other chronic diseases, such as multiple sclerosis and chronic obstructive pulmonary disease;
3. Medical complications due to diabetes or HTN, such as myocardial infarction (MI), heart failure (HF), amputation, kidney impairment, and blindness;
4. Social isolation, which is identified as having no visitors except family members and the patient not leaving the home for social activities;
5. A bedridden status;
6. Mini-Cog, a test to identify cognitive impairment;
7. Depression, identified through the geriatric depression scale;
8. Urinary incontinence: ask the patient, ‘have you ever lost urine or gotten wet?’
9. Uncontrolled diabetes: an HBA1c level of > 7%; and.
10. Uncontrolled HTN: more than 130/80 mmHg for three successive visits.

### Data analysis

Data were processed using SPSS Version 26.0 (SPSS Inc., Chicago, IL, USA). Descriptive statistics were applied as frequencies and percentages, while continuous variables such as age were reported as means and standard deviations. Additionally, logistic regression with the backward stepwise (Wald) method was used to identify predictors of frailty and polypharmacy. Model assumptions, including linearity in the logit, absence of multicollinearity, and goodness-of-fit, were checked and met. Variance inflation factors (VIFs) and tolerance statistics for all included variables were within acceptable ranges (all VIFs < 2.0; tolerance > 0.5), confirming no problematic multicollinearity.

## Results

A total of 281 CGAs for homecare patients were included in this study. A total of 82 patients (29.2%) were male while 199 (70.8%) were female. They were of a similar age. Specifically, the mean age of males was 79.56 years (SD = 8.22), while that of females was 79.70 (SD = 8.55). Both male and female patients had a median of 15 months (IQR: 9–21) since admission to homecare services. Table 1 provides the distribution of health and social indicators among participants, highlighting the prevalence of chronic conditions, frailty, polypharmacy, and other risk factors. Notably, 64.1% of respondents had both HTN and diabetes, 88.3% reported other chronic diseases, 76.5% experienced polypharmacy, 44.5% met the frailty criterion, and 47.0% reported complications. Social isolation was present in 12.5% of the respondents, and 59.4% were bedridden. Cognitive issues (Mini-Cog) affected 30.6% of respondents, depression affected 23.8%, urinary incontinence affected 27.4%, and uncontrolled HTN and haemoglobin A1C (HbA1c) affected 46.3% and 35.9%, respectively.

**Table 1:** Participant characteristics

Variable	Category	*n*	%
Age		*M* (79.70)	SD (8.55)
Gender	Male	82	29.2
	Female	199	70.8
Diabetes Miletus	No	257	91.5
	Yes	24	8.5
Hypertension	No	228	81.1
	Yes	53	18.9
Hypertension & Diabetes Miletus	No	101	35.9
	Yes	180	64.1
Other Chronic Diseases	No	33	11.7
	Yes	248	88.3
Frailty *	No	155	55.5
	Yes	125	44.5
Polypharmacy	No	66	23.5
	Yes	215	76.5
Medical Complications	No	149	53.0
	Yes	132	47.0
Social Isolation	No	246	87.5
	Yes	35	12.5
Bedridden	No	114	40.6
	Yes	167	59.4
Mini-Cog	No	194	69.0
	Yes	86	30.6
Depression	No	209	74.4
	Yes	71	25.3
Urinary incontinence	No	204	72.6
	Yes	77	27.4
Uncontrolled Blood Pressure reading	No	151	53.7
	Yes	130	46.3
Uncontrolled HbA1c	No	170	60.5
	Yes	101	35.9

* One missing value

### Frailty

A binary logistic regression was conducted to identify the predictors of frailty among older adults, and the final model demonstrated good predictive accuracy, correctly classifying 80.6% of the cases. The Hosmer–Lemeshow test was non-significant (*p* = 0.038), indicating a good fit between the observed and predicted values. The Nagelkerke R² value of 0.487 showed that the model explained more than 49% of the variance in frailty status, which is an acceptable range in clinical prediction models. Advancing age was a strong continuous predictor, with each additional year increasing the odds of frailty by 21.7% (OR = 1.217, 95% CI: 1.160–1.277, *p* < 0.001). Depression was a strong independent predictor of frailty: the odds of being frail were four times higher among those with depression than those without (OR = 0.25, 95% CI: 0.13–0.48; *p* < 0.001). For more details, see Table 2.

**Table 2:** Multivariate logistic regression analysis of factors associated with frailty (No vs Yes)

							95% C.I. For EXP(B)	
	B	S.E.	Wald	df	Sig.	Exp(B)	Lower	Upper
Constant	-15.421	2.077	55.151	1	**0.000**	0.000		
Age	0.196	0.024	64.569	1	**0.000**	1.217	1.160	1.277
DM (No Vs Yes)	− 0.049	0.559	0.008	1	0.931	0.952	0.318	2.850
HTN (No Vs Yes)	− 0.441	0.392	1.270	1	0.260	0.643	0.299	1.386
Social isolation (No Vs Yes)	0.882	0.532	2.748	1	0.097	2.417	0.851	6.860
Uncontrolled BP reading (No Vs Yes)	− 0.255	0.364	0.492	1	0.483	0.775	0.379	1.582
Depression (No Vs. Yes)	-1.387	0.335	17.156	1	**0.000**	0.250	0.130	0.482

DM: Diabetes Miletus; HTN: hypertension; Other Chronic diseases: Presence of other chronic diseases, such as multiple sclerosis and chronic obstructive pulmonary disease; Medical Complications: myocardial infarction (MI), heart failure (HF), amputation, kidney impairment, and blindness; Soc_isolation: no visitors except family members and the patient not leaving the home for social activities

Uncontrolled BP reading: more than 130/80 mmHg for three successive visits and Uncontrolled HbA1c: >7%

### Polypharmacy

A logistic regression was conducted to identify predictors of polypharmacy among older adults. The final model demonstrated acceptable predictive accuracy, correctly classifying 79.6% of the cases. The Hosmer–Lemeshow test was non-significant (*p* = 0.122), indicating a good fit between observed and predicted outcomes. The Nagelkerke R² value was 0.233, indicating that approximately 23.3% of the variance in polypharmacy status was explained by the included predictors. While this value is considered modest, it is not uncommon for behavioural and clinical models involving complex outcomes; it likely reflects the influence of additional, unmeasured factors such as healthcare access, provider practices, or medication adherence. No multicollinearity issues were detected, and the model remained stable across iterations. The final model identified key physical, psychological, and social predictors, highlighting the multifactorial nature of polypharmacy in geriatric populations.

Older adults with other chronic conditions were more than four times likely to experience polypharmacy (OR = 4.242, 95% CI: 1.822–9.876, *p* = 0.001), and those with medical complications had more than threefold increased odds (OR = 3.067, 95% CI: 1.561–6.023, *p* = 0.001). Individuals with depression also had significantly higher odds of polypharmacy (OR = 2.687, 95% CI: 1.202–6.007, *p* = 0.016). In contrast, social isolation was associated with a 73% reduction in the odds of polypharmacy (OR = 0.273, 95% CI: 0.118–0.634, *p* = 0.003).

Similarly, uncontrolled HbA1c was associated with lower odds (OR = 0.559, 95% CI: 0.319–0.982, *p* = 0.043), and DM also emerged as a protective factor (OR = 0.319, 95% CI: 0.122–0.834, *p* = 0.020). For more details, see Table 3.

**Table 3:** Multivariate logistic regression analysis of factors associated with polypharmacy (No vs. yes)

							95% C.I. for EXP(B)	
	B	S.E.	Wald	df	Sig.	Exp(B)	Lower	Upper
Constant	-4.276	1.937	4.876	1	0.027	0.014		
Age	0.029	0.019	2.520	1	0.112	1.030	0.993	1.068
DM (No Vs Yes)	-1.141	0.490	5.436	1	**0.020**	0.319	0.122	0.834
HTN (No Vs Yes)	0.782	0.466	2.813	1	0.094	2.186	0.876	5.451
Other Chronic (No Vs Yes)	1.445	0.431	11.237	1	**0.001**	4.242	1.822	9.876
Medical Complications ( No vs. Yes)	1.121	0.344	10.587	1	**0.001**	3.067	1.561	6.023
Social isolation (No Vs Yes)	-1.297	0.429	9.135	1	**0.003**	0.273	0.118	0.634
Uncontrolled HbA1c (No Vs Yes)	− 0.581	0.287	4.091	1	**0.043**	0.559	0.319	0.982
Depression (No Vs. Yes)	0.988	0.410	5.799	1	**0.016**	2.687	1.202	6.007

DM: Diabetes Miletus; HTN: hypertension; Other Chronic diseases: Presence of other chronic diseases, such as multiple sclerosis and chronic obstructive pulmonary disease; Medical Complications: myocardial infarction (MI), heart failure (HF), amputation, kidney impairment, and blindness; Soc_isolation: no visitors except family members and the patient not leaving the home for social activities

Uncontrolled BP reading: more than 130/80 mmHg for three successive visits and Uncontrolled HbA1c: >7%

## Discussion

The current study explored the epidemiological profiles of geriatric home care patients in Madinah, Saudi Arabia. The results showed a significant burden of chronic disease among the study sample of homecare patients. Such a burden may reflect the increasing level of home care services required to meet patients’ healthcare needs and provide proper support. This burden may impact healthcare costs, patients’ health, and caregivers’ need for support.

Consistent with earlier studies, diabetes and HTN were the most frequently identified comorbidities in the current study. However, compared to prior studies, our results showed lower percentages for DM and HTN when estimated as stand-alone diseases. Conversely, the estimated prevalence of both diseases, when they occurred simultaneously, was higher in the current study than in previous studies [6–21, 23]. These differences could be attributed to the older age of the current study sample, with medical complications due to chronic diseases typically increasing with age.

Differences were also evident in other domains, such as depression and urinary incontinence, for which the current study showed higher levels of prevalence than prior studies. Therefore, the researcher argues that comparisons between the outcomes of the current study and earlier ones, considering the predominance of the older population (79 years old), may indicate the real burden of chronic diseases and the need for an expansion of home care services to meet patients’ needs in the coming decade.

In the current study, the prevalence of frailty was high among the participants. This rate was notably higher than the prevalence reported in a community-based study of individuals aged 60 years or older across Saudi Arabia [14]. The elevated prevalence in the current study may be attributable to the specific characteristics of home care patients, who often have multiple comorbidities and functional impairments necessitating comprehensive care. Additionally, a systematic review and meta-analysis focusing on Middle Eastern countries reported a wide range of frailty prevalence rates among individuals aged 60 and older [15]. Globally, frailty prevalence varies widely depending on the setting and assessment tools [16, 17]. Moreover, physical frailty was higher in females than in males in a review of 62 countries [18]. These findings suggest the influence of regional, methodological, and population-specific factors on frailty prevalence [15]. The higher prevalence observed in our study highlights the critical need for further assessments, targeted interventions, and resource allocation to address frailty among homecare patients in the Al Madinah region.

It is important to mention that the operational definition of frailty used in the current study, derived from the CGA and used at the study location (85 years or older), does not match the definitions used in the aforementioned studies, which were based on the Edmonton Frail Scale and Fried’s frailty index [24]. Frailty can affect people regardless of age, as several contributing factors, such as diseases, can exacerbate it. Interestingly, in the current study, several participants who experienced severe medical complications, such as HF, amputation, or kidney impairment, were classified as non-frail solely because they were younger than 85 years. This suggests that the current age-based frailty criterion may overlook clinically vulnerable individuals, highlighting the need for a more comprehensive definition that incorporates functional and clinical parameters. The study findings encourage the Saudi MoH’s homecare administration to update the operational definition of frailty to align with international recommendations, as frailty cannot be adequately assessed using age alone. The current study supports previous calls to adopt multidimensional frailty screening tools in geriatric assessments [25, 26].

The prevalence of polypharmacy in the current study aligns with the findings of a recent Saudi study, particularly among the oldest age group (> 79 years) [27]. However, the results indicate a higher prevalence than in other national and international studies [6, 12, 13]. These differences could be attributed to the older age of the current study sample and the complexity of their medical conditions, which exacerbate the burdens of polypharmacy [13].

It was noticed in the polypharmacy model that the observed protective associations for diabetes (OR = 0.319) and uncontrolled HbA1c (OR = 0.559) were unexpected and contrary to clinical expectations. These counterintuitive findings may be attributable to survivor bias, unmeasured confounding factors (e.g., differences in medication adherence, healthcare utilisation, or disease severity), or limitations in how polypharmacy and glycaemic control were operationalised in this analysis. As such, these results should be interpreted with caution, and further research, including longitudinal studies with more detailed clinical data, is warranted to clarify the underlying mechanisms.

In general, the polypharmacy model showed that the presence of other chronic conditions, medical complications, depression, and hypertension increases the likelihood of polypharmacy. Therefore, the current study encourages homecare decision makers to implement targeted strategies aimed at optimising medication utilisation. These could include routine pharmacist-led medication reviews, application of deprescribing protocols (e.g., STOPP/START criteria), use of medication reconciliation tools, and integration of telepharmacy services to support remote deprescribing [28, 29]. Such interventions focus on minimising the inappropriate use of medications and adverse drug events without compromising the management of chronic conditions. Given the high disease burden among this population, the goal should not be to reduce medication count arbitrarily but to ensure clinical appropriateness, safety, and patient-centred medication management.

From a financial burden perspective, frailty and polypharmacy are associated with substantial healthcare costs due to increased hospital admissions, prolonged care needs, and adverse drug events [30]. Frailty increased hospital admission costs at follow-up by 24% per year compared with non-frail individuals [31]. Moreover, frailty alone can add more than $10,000 in annual healthcare costs per patient [32]. However, avoidable costs associated with polypharmacy among the elderly amount to $1.3 billion annually [33]. To reduce this burden, policy measures should focus on integrating pharmacist-led medication reviews, applying deprescribing protocols, and utilising clinical tools such as STOPP/START criteria to optimise prescribing practices in older adults.

### Implications for practice

This study sheds light on the profiles of geriatric homecare patients, revealing high levels of frailty and polypharmacy. A multidisciplinary approach by the homecare team that includes physicians, nurses, pharmacists, clinical dietitians, and social specialities is recommended to optimise medication regimens and reduce the risks associated with polypharmacy. More importantly, updating the current definitions of frailty and polypharmacy to align with evidence-based international practice could enhance the accuracy of CGAs and, consequently, the quality of home care.

From a policy standpoint, this study suggests three actionable recommendations. First, a universal frailty screening protocol should be applied in homecare settings. Adapting validated tools such as the Edmonton Frail Scale may enhance early detection and management of frailty. Second, implement scheduled medication review intervals to minimise inappropriate prescribing and adverse drug interactions. Third, invest in training homecare nurses in polypharmacy risk-reduction strategies. These actions can be easily implemented taking into account the existing strong homecare infrastructure, especially for those under MoH, presence of multidisciplinary teams, and support by the Saudi Commission for Health Specialities, which imposes continuing education modules and awards diplomas for homecare nurses. Finally, nursing researchers should support the expansion of homecare research and the best evidence-based approaches utilisation.

While the age-based definition used in this study reflects local practice, it likely underrepresents the true burden of frailty, as it overlooks younger individuals with significant clinical vulnerability. This reinforces the need to adopt validated tools, such as the Fried or Edmonton scales, in future research and routine homecare assessments.

### Strengths and limitations

This study has various limitations. Its cross-sectional design prevents the identification of causality. Although the study reached the optimum sample size, taking into account time and logistic restraints, the generalisability of the results may still be limited. Utilising CGAs to extract data from practice was a strength, but the differing definition of frailty from those used in international studies limits the generalisability of the results to broader populations, as younger, clinically vulnerable individuals were not included in the definition. Data collection was conducted systematically by authorised healthcare team members, which maintained the confidentiality of patient information and increased data accuracy. Finally, the study focused on a high-priority area (frailty and polypharmacy among elderly adults), which can help address key health challenges and inform healthcare policy.

## Conclusion

The study revealed that frailty was predicted by increased age, depression, and diabetes, while polypharmacy was predicted by the presence of other chronic conditions, medical complications, depression, and hypertension. Despite the imperative need for homecare to improve the management of these diseases, the study findings are limited by frailty’s operational definition and the retrospective cross-sectional study design’s inherited limitations.

The findings highlight the need for tailored healthcare interventions to address frailty and polypharmacy in homecare settings. Tailored multidisciplinary interventions, including pharmacist-led medication reviews, are essential to improve health outcomes and medication safety in this growing population. Moreover, the study recommends that future research explore universal operational definitions of frailty in national practice to better align with global standards.

## Data Availability

The datasets used and/or analysed during the current study are available upon reasonable request.
